# 49 Autoimmune hemolytic anemia revealing a familial systemic lupus erythematosus

**DOI:** 10.1093/rheumatology/keac496.045

**Published:** 2022-10-07

**Authors:** Rouag Hatem, Zayani Seif, Akid Alaa, Sfar Emna, Ghedira Tesnime, Hadj Salem Radhia, Chouchane Chokri, Thabet Farah, Chouchane Slah

**Affiliations:** Department of Pediatrics, Fattouma Bourguiba Hospital Monastir, Tunisia

## Abstract

**Background:**

Systemic lupus erythematosus (SLE) is a heterogeneous autoimmune disease; whose pathogenesis remains poorly understood. The genetic susceptibility to lupus is supported by the high concordance rate of the disease in monozygotic twins (29–57%) compared with dizygotic twins (2%), and by the existence of familial lupus (10–12%). Furthermore, SLE shares genetic and environmental risk factors, as well as pathophysiologic mechanisms with autoimmune hemolytic anaemia (AIHA), which is clearly over-represented in patients with SLE and often occurs before the diagnosis of the underlying disease.

**Objective:**

We report a case of familial SLE in two siblings, in whom, the main symptom was AIHA.

**Case report:**

A twelve-year-old girl was referred to our Pediatric department for polyarthralgia, asthenia and anorexia developing over the previous 4–6 months. Regarding family history, she is born of a non-consanguineous marriage and has a 7-year-old brother who has been followed for 2 years for AIHA of undetermined aetiology. The examination upon admission objectified a splenomegaly and jaundice. The laboratory workup revealed an autoimmune hemolytic anaemia: haemoglobin: 7.2 g/dl, positive Coombs' test with specific anti globulin IgG and C3d; unconjugated bilirubin (45 µmol/l) and reduced haptoglobin (0.01 g/l). These findings being very evocative of SLE, an antinuclear antibody (AAN) assay was positive at 1/400. The formal SLE diagnosis was established according to the EULAR/ACR 2019 criteria; with a score of 12 (AIHA: 4; positive antiphospholipid antibodies: 2; positive anti-Sm antibodies: 6). The therapeutic management based on corticosteroids at a dose of 1 mg/kg/day, with a favorable clinical and biological outcome. In light of this diagnosis, the case of the aforementioned brother was reviewed, and a familial lupus was suspected. A physical examination and a several biological explorations were done, the results of which allowing to retain the diagnosis of SLE with EULAR/ACR 2019 score of 10 (ANA > 1/80, AIHA : 4, positive anti-phospholipid antibodies: 2, proteinuria: 4)

**Conclusion:**

Since the new criteria EULAR/ACR 2019 have made it possible to diagnose incomplete forms of lupus, the challenge remains to detect autoimmune thyroiditis, antiphospholipid syndrome, complement deficiencies and other entities that are associated with familial lupus according to scientific literature.

